# Anti‐Inflammatory and Lipid Metabolism Modulatory Effects of *Lantana camara* L. Extract, With Enhanced Fibroblast Migration

**DOI:** 10.1002/cbdv.202501335

**Published:** 2025-12-15

**Authors:** Ari S. de O. Lemos, Matheus T. Branca, Lara M. Campos, Natasha S. Mayrink, Lívia R. Gamarano, João Pedro R. C. Bastos, Ana Julia de Almeida, Elaine S. Coimbra, Rodrigo L. Fabri

**Affiliations:** ^1^ Bioactive Natural Products Laboratory, Department of Biochemistry Institute of Biological Sciences, Federal University of Juiz de Fora Juiz de Fora Brazil; ^2^ Department of Parasitology, Microbiology, and Immunology Institute of Biological Sciences, Federal University of Juiz de Fora Juiz de Fora Brazil

**Keywords:** Cambará, complex inflammatory process, cytokines, nitric oxide, wound healing

## Abstract

Inflammation is a complex process associated with various chronic diseases. *Lantana camara* L., widely used in traditional medicine, exhibits diverse bioactivities, though its effects on lipid metabolism, prostaglandin E2 (PGE2) production in macrophages, and fibroblast migration remain unclear. This study aimed to evaluate whether the ethanolic extract from *L. camara* leaves (LCE) influences these processes. Phytochemical analysis by an ultra‐fast liquid chromatography‐quadrupole time of flight mass spectrometry identified flavonoids and iridoid glycosides in the extract. Cytotoxicity assays using BALB/c mouse peritoneal macrophages and L929 fibroblasts showed no significant effect on cell viability. Anti‐inflammatory activity was assessed through nitric oxide (NO), reactive oxygen species (ROS), lipid bodies (LB), and levels of tumor necrosis factor (TNF)‐α, transforming growth factor (TGF)‐β, and PGE2. LCE significantly reduced NO (74.61 ± 5.08%), ROS (58.38 ± 1.77%), and LB formation (53.56 ± 3.33%), as well as TNF‐α (40.63 ± 1.33%), TGF‐β (88.96 ± 12.87%), and PGE2 (56.88 ± 27.10%) levels. Wound healing potential was evaluated via scratch assay, with LCE promoting fibroblast migration up to 86.26 ± 0.81% after 48 h. These findings indicate that LCE modulates macrophage inflammatory responses and promotes tissue repair, supporting its potential as a natural therapeutic agent for managing inflammation, oxidative stress, and wound healing.

## Graphical abstract

This graphical abstract represents the main results of the study. It highlights the plant species under investigation: *Lantana camara* L.

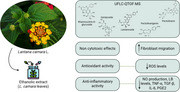

## 1 Introduction

Production of radicals can be derived from external sources such as exposure to air pollutants, X‐rays, alcohol and cigarette consumption, exposure to heavy metals, transition metals, and industrial chemicals. However, the metabolic processes of organs present in human cells, such as peroxisomes, mitochondria, and endoplasmic reticulum, can also produce these mediators [1, 2]. In low concentrations, free radicals function in physiological cellular processes, but their excessive production causes damage to cellular components such as lipids, proteins, and DNA [3].

Inflammation is a complex process with many events linked to chronic diseases like diabetes, heart disease, and cancer. It is also tied to oxidative stress through the creation of reactive oxygen and nitrogen species [4]. In this process, cyclooxygenase‐2 (COX‐2) is an essential enzyme that is activated by a number of different substances, including arachidonic acid, lipopolysaccharide (LPS), interleukin‐1 (IL‐1), tumor necrosis factor (TNF), epidermal growth factor (EGF), and platelet‐activating factor (PAF) [5]. Lipid bodies (LBs) are dynamic organelles that have a role in inflammation and lipid metabolism. It has been established that COX‐2 is present in LBs, indicating that these organelles are eicosanoid synthesis sources [6].

Based on their effects on COX‐2, several pharmacological classes are very successful in treating inflammation; nevertheless, these medications come with a host of adverse effects [7]. Therefore, the discovery of new anti‐inflammatory therapies has been of great interest. Among the alternative therapeutic options, we can mention the use of natural products, since they can present anti‐inflammatory therapeutic effects and have low side effects [8]. *Lantana camara* L. is a plant with a broad spectrum of bioactivities. In traditional medicine, *L*. *camara* has also been used to treat a variety of inflammatory illnesses, including rheumatism and bone pain, using its leaves, roots, and flowers [9]. Regarding the anti‐inflammatory activity, the leaves’ ethanolic extract (LCE) has shown COX‐2 inhibitory effects and reduced inflammatory cytokines [10]. Furthermore, flavonoids, phenolic substances with the ability to regulate many phases of inflammation, are thought to be connected to the anti‐inflammatory activity of *L*. *camara* [11].

Inflammation and wound healing are intricately linked processes. Moderate inflammation promotes wound healing, whereas excessive inflammation causes scarring, delayed wound healing, and perhaps serious pathological alterations. [12] The early stages of healing involve inflammation, where immune cells like macrophages and neutrophils are recruited to the site of injury. These cells release pro‐inflammatory cytokines and growth factors that stimulate fibroblasts to proliferate, migrate, and synthesize extracellular matrix components such as collagen, which are essential for tissue repair. However, an imbalance between pro‐inflammatory and anti‐inflammatory signals can lead to impaired wound healing [12].

Although previous studies have reported the anti‐inflammatory properties of *Lantana camara* extracts, the mechanism of action of *L. camara* on lipid metabolism, prostaglandin E2 (PGE2) production in macrophages, and fibroblast migration has not yet been elucidated. Therefore, this work is innovative in integrating these aspects to evaluate the potential of LCE to modulate inflammatory lipid mediators and promote fibroblast migration during wound healing, providing new insights into its therapeutic applications.

## 2 Results and Discussion

Five compounds were found, as listed in Table 1, after the chromatographic profile was examined using an ultra‐fast liquid chromatography‐quadrupole time of flight mass spectrometry (UFLC‐QTOF‐MS) in order to identify the main compounds present in LCE. LCE showed mainly flavonoids and iridoid glycosides in its composition, which is in agreement with the data found in the literature, which states that the species possesses a large amount of these substances, which have been shown to exhibit biological activity [13, 14].

**TABLE 1:** Compounds in leaves’ ethanolic extract (LCE) detected in electrospray ionization (ESI) (+) by an ultra‐fast liquid chromatography‐quadrupole time of flight mass spectrometry (UFLC‐QTOF‐MS).

Substance classes	Substance	Molecular structure	Rt (Min)	Mass fragmentation	References
Flavonoid	Rhamnocitrin‐O‐glucoside	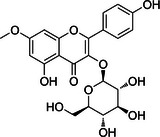	3.9	480.2087 (M+NH_4_)	[13]
Iridoid glycosides	Geniposide	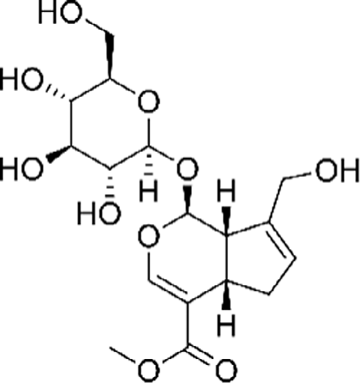	5.8	799.4108 (2 M+Na); 411.1996 (M+Na)	[13]
Flavonoid	Lantanoside	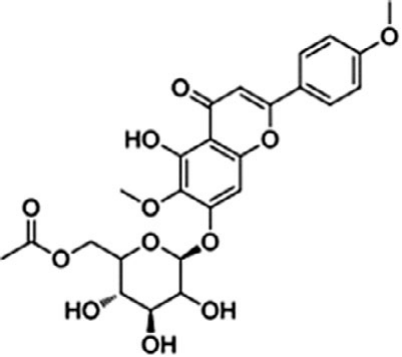	6.4	541,2288 (M+Na); 467,1557 (M+Na ‐CO_2_‐2CH_3_)	[14]
Flavonoid	Pectolinarigenin	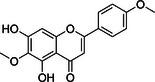	7.1	337,0900 (M+Na); 153,0548 (M+Na ‐2CO_2_ ‐ 2H_2_O ‐ 2CH_2_O)	[14]
Flavonoid	Pectolinarin	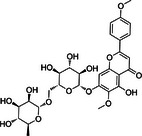	9.1	640,2236 (M+NH4); 503,2115 (M+NH4 ‐ C_8_H_9_O_2_)	[14]

Rt, Retention time.

**FIGURE 1:**
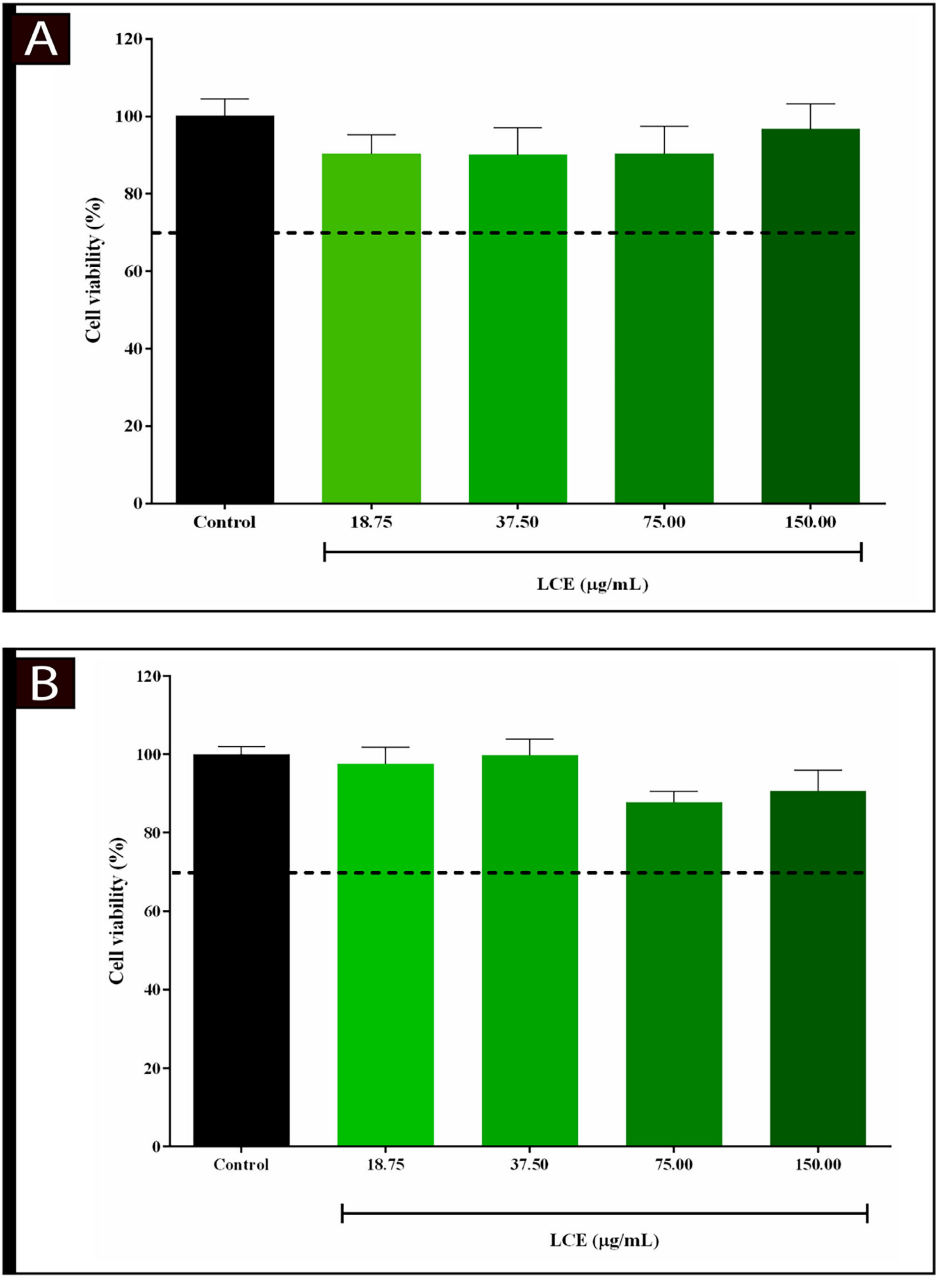
Evaluation of cell viability in BALB/c mouse macrophages (A) and L929 fibroblasts (B) treated with ethanolic extract of *L. camara* leaves (LCE) using the MTT assay. Results were expressed as mean ± standard deviation of three independent experiments. One‐way analysis of variance (ANOVA) followed by Dunnett's Multiple Comparison post hoc test. ^a^Statistical difference when compared to control (*p* < 0.05).

The LCE extract was evaluated for cytotoxicity in macrophage and fibroblast cells according to the criteria of the ISO 10993‐5:2009 standard, which regulates in vitro tests for the evaluation of cytotoxicity. According to this standard, a substance is considered safe for use if cell viability is greater than 70% [15]. The results obtained showed that cell viability under the conditions of exposure to the LCE extract was above this threshold, indicating reduced toxicity. Furthermore, statistical analysis revealed no significant differences (*p* > 0.05) between the experimental groups, confirming the absence of cytotoxic effects on the cells tested. Figures 1A and 1B clearly illustrate these results and show the absence of significant cellular changes. These results suggest that the LCE extract has no cytotoxic potential under the experimental conditions analyzed, thus meeting established safety standards.

**FIGURE 2:**
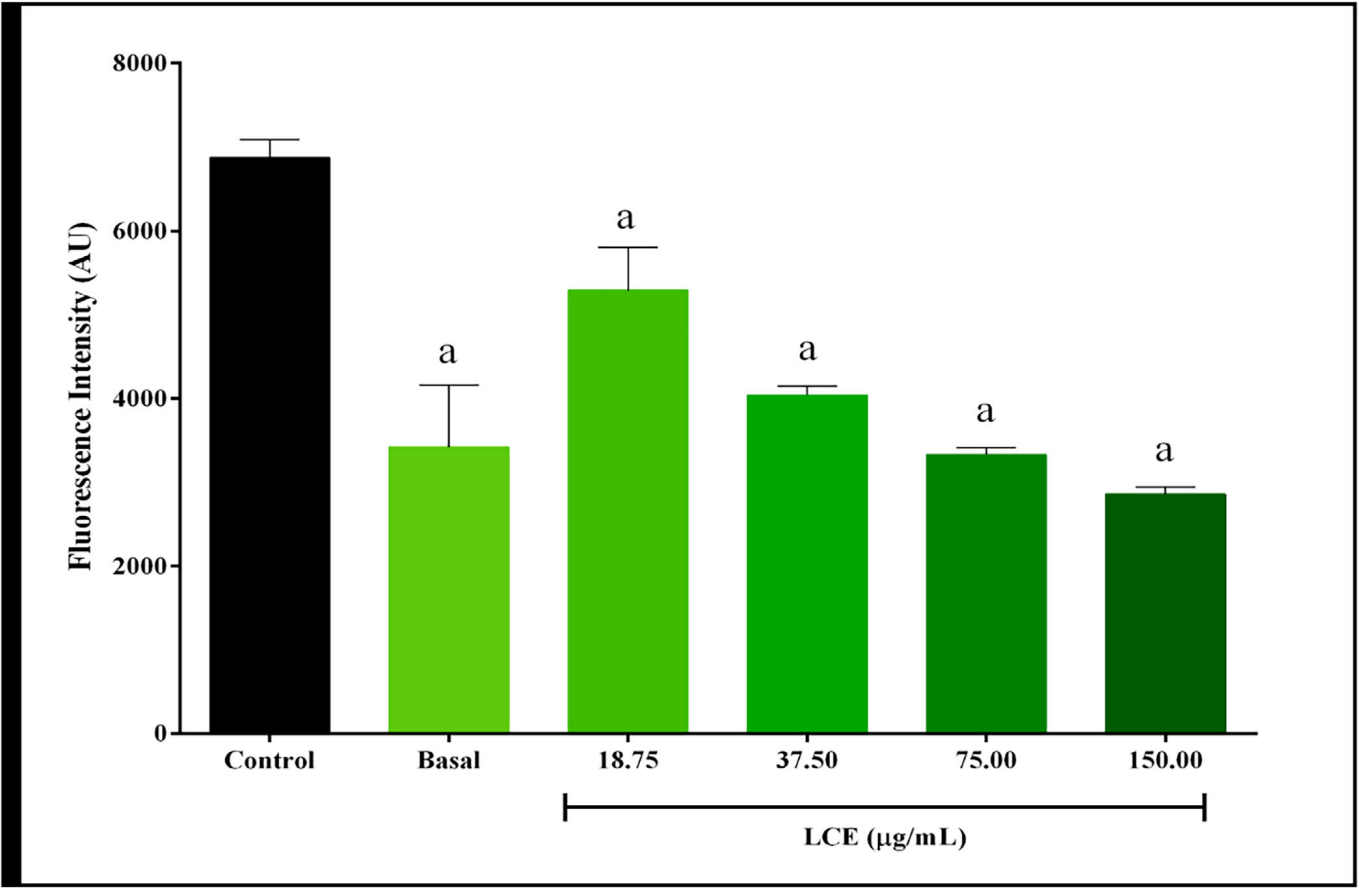
Reactive oxygen species (ROS) production in peritoneal macrophages stimulated with LPS at 1 µg/mL and interferon (IFN)‐γ at 1 ng/mL and treated with leaves’ ethanolic extract (LCE) using H_2_DCFDA. Control—Cells stimulated with lipopolysaccharide (LPS) and IFN‐γ. Results were expressed as mean ± standard deviation of three independent experiments. One‐way analysis of variance (ANOVA) followed by Dunnett's Multiple Comparison post hoc test. ^a^Statistical difference when compared to control (*p* < 0.05). Equal letters indicate no significant difference between groups.

Different compounds derived from molecular oxygen are known as reactive oxygen species (ROS) [16], which play a crucial role in physiological cellular processes, but are also associated with cell damage and the development of various pathologies when present in high concentrations. The antioxidant activity of compounds was evaluated in cells stimulated with LPS and IFN‐γ, which induce an inflammatory environment and increase ROS production. For this purpose, ROS levels were quantified. The results showed that LCE extract was able to significantly reduce ROS levels compared to the control at all concentrations tested (Figure 2, *p* < 0.05). These results suggest that LCE exerts a protective effect against oxidative stress, possibly due to its antioxidant activity, which can neutralize free radicals and other reactive oxygen species, thereby mitigating cell damage resulting from induced oxidative stress. In addition, the observed reduction in ROS levels (Inhibition of 58.38 ± 1.77 and 51.56 ± 1.69 at concentrations of 150.00 and 75.00 µL, respectively) may be related to LCE's ability to modulate the activation of inflammatory pathways such as COX‐2, an enzyme involved in the synthesis of pro‐inflammatory prostaglandins. Inhibition of COX‐2 can lead to a reduction in free radical production and control of oxidative stress. In this way, LCE not only exerts a direct antioxidant effect but can also act as a modulator of inflammatory pathways, providing an additional protective effect to cells exposed to oxidative stress.

**FIGURE 3:**
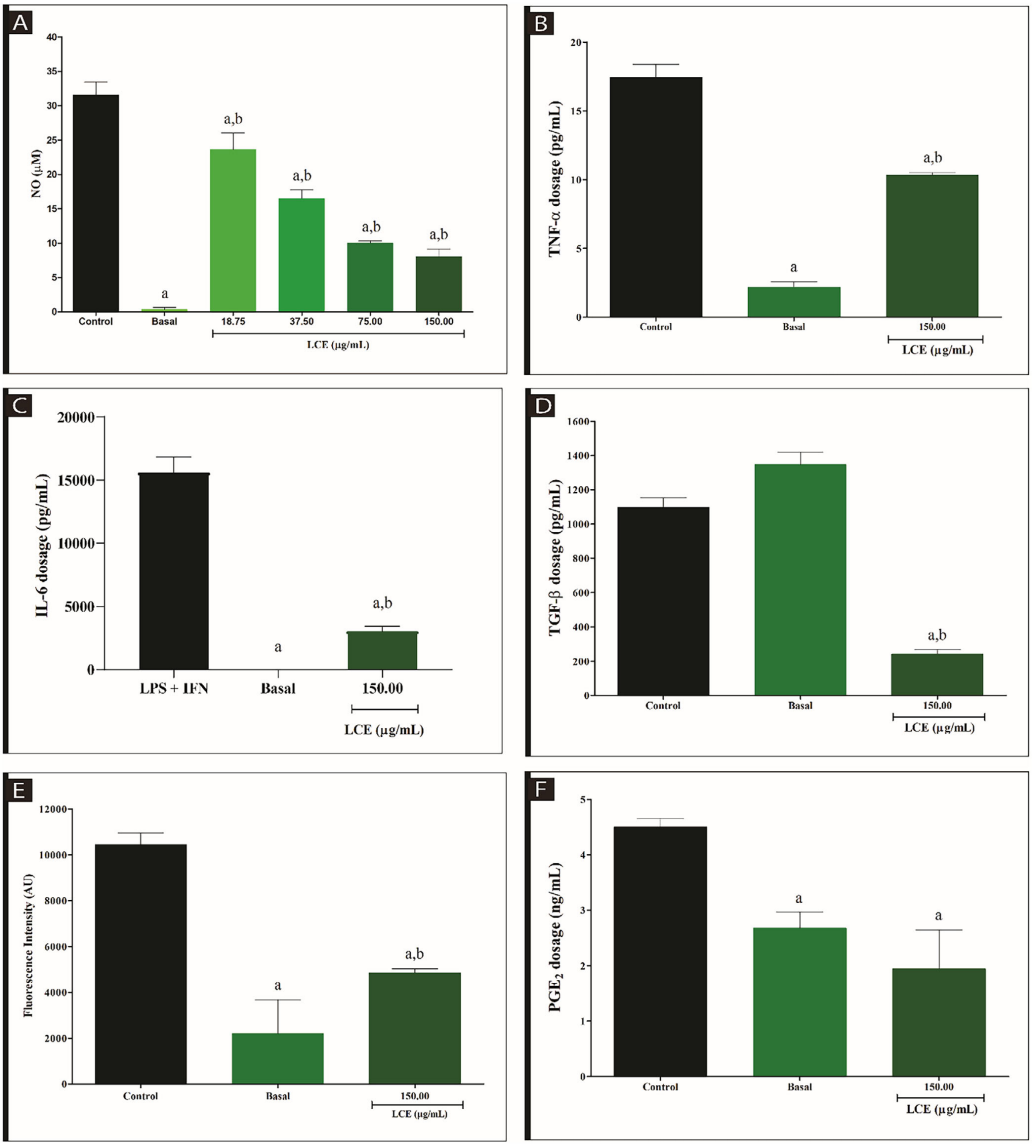
Evaluation of the anti‐inflammatory activity of the ethanolic extract from *L. camara* leaves (LCE). (A) Nitric oxide (NO) production in macrophages obtained from BALB/c mice stimulated with LPS at 1 µg/mL and interferon (IFN)‐γ at 1 ng/mL and treated with LCE by the Griess method. (B) Dosage of tumor necrosis factor (TNF)‐α levels produced in peritoneal macrophages treated with LCE by enzyme‐linked immunosorbent assay (ELISA). (C) Dosage of interleukin (IL)‐6 levels produced in peritoneal macrophages treated with LCE by ELISA assay. (D) Dosage of transforming growth factor (TGF)‐β levels produced in peritoneal macrophages treated with LCE by ELISA assay. (E) Evaluation and Quantification of lipid bodies (LBs) accumulated using Nile Red. (F) Dosage of PGE_2_ levels produced in peritoneal macrophages treated with LCE by ELISA assay. Control—Cells stimulated with LPS and IFN‐γ. Results were expressed as mean ± standard deviation of three independent experiments. One‐way analysis of variance (ANOVA) followed by Dunnett's Multiple Comparison post hoc test. ^a^Statistical difference when compared to control (*p* < 0.05). ^b^Statistical difference when compared to the basal group.

The potential of LCE extract to attenuate the production of nitric oxide (NO), a free radical, was demonstrated in our study, suggesting a possible modulatory pathway in the activation of COX‐2. NO is widely recognized as a key intermediate in the inflammatory response and is involved in the activation of COX‐2, a critical enzyme for the production of inflammatory prostaglandins [17]. To assess the production of NO, we measured nitrite levels, which are the end products of NO oxidation. The results obtained showed a statistically significant reduction in nitrite levels (p < 0.05) in the concentrations of LCE tested (37.5 ‐ 150 µg/mL) compared to the control group (Inhibition of 74.61 ± 5.08 and 68.30 ± 1.58 at concentrations of 150.00 and 75.00 µL respectively), except for the concentration of 18.75 µg/mL, which showed no significant differences (Figure 3A). These results suggest that LCE may exert an antioxidant effect by reducing NO production, which may contribute to the inhibition of COX‐2 activation and consequently reduce the associated inflammation. These results indicate that LCE has properties that may attenuate free radical generation, providing a potential approach to control inflammatory processes mediated by COX‐2.

The cytokine TNF‐α plays a fundamental role in various cellular processes such as cell death, differentiation, proliferation, and survival. It is mainly produced by cells of the immune system, such as macrophages and T lymphocytes, and acts as a critical mediator in the inflammatory response [18]. TNF‐α is known for its ability to activate signaling pathways that regulate the expression of several inflammatory molecules, including COX‐2 [19]. In this context, the LCE extract showed significant anti‐inflammatory potential. Treatment with LCE resulted in a significant reduction in TNF‐α levels, indicating its ability to interfere with inflammatory pathways, probably by inhibiting TNF‐α production (Inhibition of 40.63 ± 1.33) (Figure 3B; *p* < 0.05). In addition, LCE was also able to reduce IL‐6 production after stimulation with LPS and IFN (Inhibition of 80.62 ± 2.47), demonstrating its ability to modulate inflammatory pathways mediated by multiple cytokines (Figure 3C; *p* < 0.05). These results suggest that LCE has the potential to interfere with different stages of the inflammatory cascade, providing an effective therapeutic strategy for inflammatory conditions.

In addition, TGF‐β, a cytokine widely known for its involvement in the formation of LBs, was investigated [20, 21]. The results obtained showed that the production of TGF‐β was significantly reduced after treatment with LCE (Inhibition of 88.96 ± 12.87%) (Figure 3D; *p* < 0.05), suggesting that LCE exerts an inhibitory effect on the formation of LBs and interferes with the regulatory pathways associated with this inflammatory mediator.

LBs, dynamic and conserved structures in all cell types, are essential for the storage of intracellular lipids [22]. Literature data indicate that treatment with LPS promotes increased formation of LBs and increases PGE2 levels in leukocytes isolated from the pleural cavity of mice [23]. Consistent with previous studies, an increase in LB levels was observed in the control group. However, LCE treatment resulted in a significant reduction in LB formation compared to the control group (Inhibition of 53.56 ± 3.33 %) (Figure 3E; *p* < 0.05), suggesting that LCE may negatively modulate LB biogenesis. In particular, LCE treatment also resulted in a significant decrease in PGE2 levels (Inhibition of 56.88 ± 27.10) (Figure 3F; *p* < 0.05), reinforcing the hypothesis that LCE negatively affects both the biosynthesis of LBs and the production of related inflammatory mediators such as PGE2.

**FIGURE 4:**
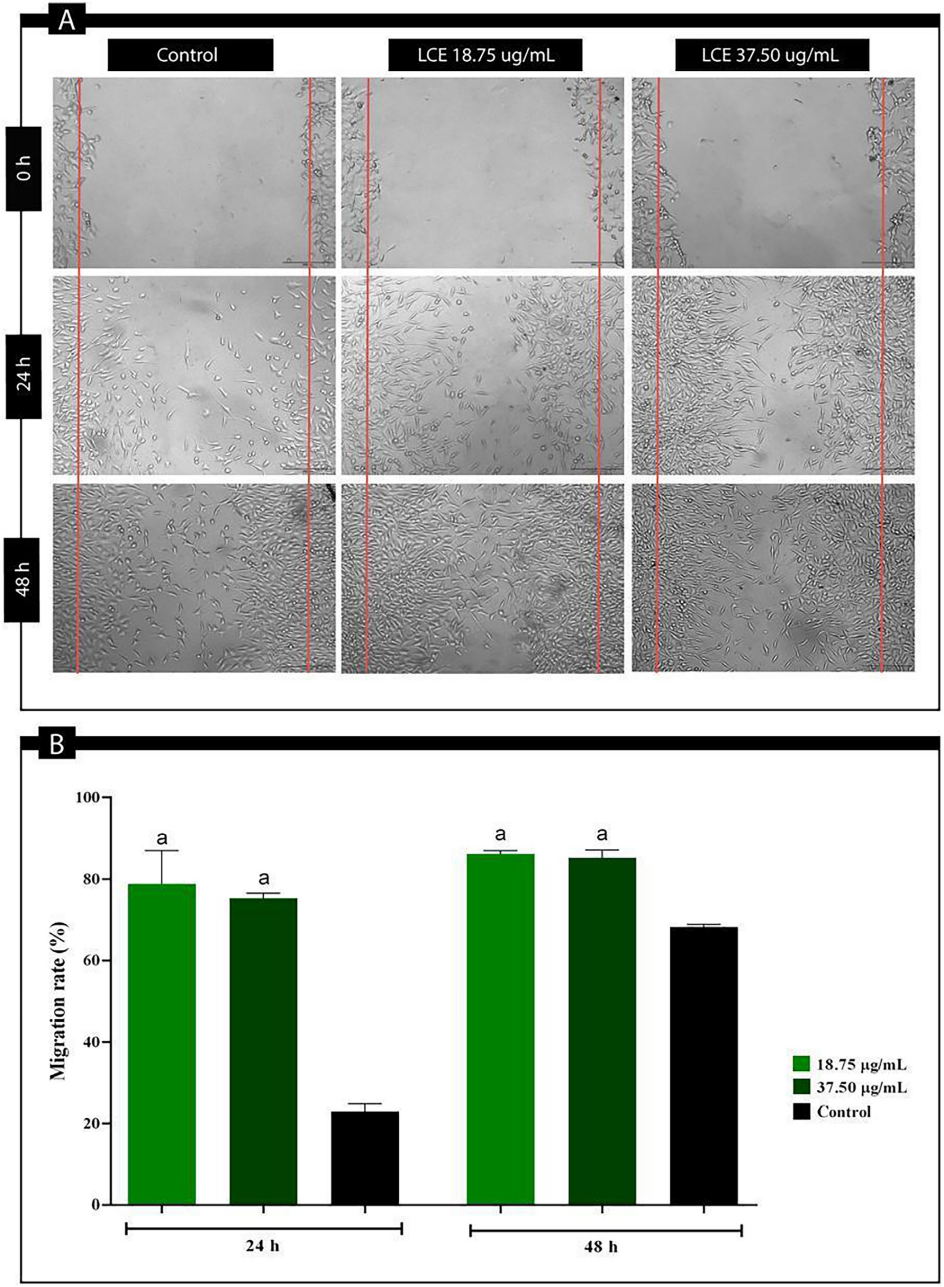
Effect of *L. camara* leaves ethanolic extract (LCE) on L929 fibroblast migration. (A) Images of L929 fibroblasts treated with LCE (18.75 and 37.50 µg/mL) and the control group at 0, 24, and 48 h after scratch. (B) Migration rate of L929 fibroblasts measured 24 and 48 h after scratching. ^a^Statistical difference when compared to control (p < 0.05). Equal letters indicate no significant difference between groups.

On the other hand, fibroblast migration and proliferation play a critical role in collagen production and cellular matrix formation [24, 25]. Given the importance of these cells in the healing process, compounds that enhance their migration and proliferation may significantly improve tissue repair [25]. To simulate the process of fibroblast migration, the scratch assay was performed. The results showed that LCE at both concentrations tested (18.75 and 37.50 µg/mL) significantly accelerated the proliferation of L929 fibroblasts compared to the control group (Figure 4A; *p* < 0.05). After 24 h, treatment with LCE at 18.75 µg/mL resulted in 78.82 ± 8.22% cell migration, whereas at 37.50 µg/mL the migration was 68.60 ± 11.58%. After 48 h, LCE at 18.75 µg/mL stimulated 86.26 ± 0.81% of cell migration, while at 37.50 µg/mL it stimulated 79.88 ± 9.54%. These results show that LCE is more effective in promoting cell migration than the control group (24 h: 27.24 ± 6.68%; 48 h: 71.29 ± 4.76%), especially at the 18.75 µg/mL concentration (Figure 4B; *p* < 0.05).

The leaves of *L. camara* are widely used in traditional medicine for the treatment of wounds [26]. In a study by Sultana et al. [27], an emulgel containing *L. camara* leaf extract accelerated wound contraction and reduced epithelization time in infected rat wounds, which was statistically similar to the standard group. According to Samal et al. [28], the aqueous extract of *L. camara* flowers also showed significant wound healing activity in wound models after increasing the wound contraction rate. In addition, Vaishnav et al. [29], using silver nanoparticles loaded with the aqueous extract of *L. camara* leaves, observed that the formulation significantly reduced the *in vitro* wound area after 24 h.

The results presented here may be related to the prominent anti‐inflammatory and antioxidant activities, as well as the rich flavonoid composition of *L. camara* leaves. According to the literature, the presence of compounds such as flavonoids, alkaloids, and tannins is closely associated with wound contraction and epithelialization. [29, 30] Flavonoids, such as lantanoside, pectolinarigenin, and pectolinarin, identified in LCE, have also been reported to be effective in the treatment of diabetic wounds. [30].

The results previously shown can be attributed to specific compounds present in LCE. The anti‐inflammatory activity of *Lantana camara* has been extensively studied and is attributed to the presence of bioactive compounds such as flavonoids and iridoids [31, 32]. The flavonoids are known for their potent inhibition of arachidonic acid, phospholipase A2, cyclooxygenase, and iNOS. This reduces the production of prostaglandins, leukotrienes, and NO, which are key inflammatory substances [33]. Pectolinarigenin possesses the ability to reduce NO in macrophages and decrease the release of inflammatory mediators, IL‐1β, and IL‐6 induced by LPS. In addition, this flavonoid also has the ability to suppress NFκB in astrocyte culture induced by LPS [34]. Geniposide, in turn, is an iridoid glycoside that can decrease the levels of IFN‐γ, IL‐6, IL‐17, and other inflammatory cytokine levels and increase the level of anti‐inflammatory cytokines such as TGF‐β1, IL‐4 in which are secreted by fibroblast‐like Synoviocytes or mesenteric lymph node [35]. Furthermore, the Pectolinarigenin has been shown to inhibit COX‐2‐mediated PGE2 synthesis from LPS‐treated RAW 264.7 cells, leading to reduced eicosanoid production. In addition, oral administration of pectolinarigenin and pectolinarin has also been known to inhibit animal models of inflammation and allergy [36].

## 3 Conclusions

LCE has minimal toxicity and can modulate lipid metabolism in macrophages activated by LPS and IFN‐γ, without compromising the integrity of cell membranes. The reduction in PGE2 and TGF‐β levels is in line with the reduction in the formation of lipid bodies, demonstrating its anti‐inflammatory effect. In addition, LCE was able to reduce ROS and NO levels, reinforcing its antioxidant potential. L929 fibroblast migration and wound closure rates were also significantly improved. These findings suggest that LCE is a multifaceted anti‐inflammatory agent, with action on various inflammatory pathways, probably mediated by its chemical composition, highlighting flavonoids and irinoid glycosides.

## 4 Experimental

### 4.1 Plant Material

*Lantana camara* L. leaves were collected in Juiz de Fora, state of Minas Gerais, Brazil (coordinates 21°77’62“ south, 43°37’05” west), in January 2019, according to the license number A18AB08 SISGEN/BRAZIL. A voucher specimen (CESJ 30671) was deposited at the CESJ Herbarium.

### 4.2 Preparation of the LCE

LCE was obtained after static maceration of 90 g of dried and previously pulverized leaves in ethanol P.A. (Dinâmica Química Contemporânea Ltda, Brazil). After extraction (3 x 500 mL), the resulting liquid was concentrated at reduced pressure using a rotary evaporator (Laborota 4000, Heidolph, Germany). After this step, the extract was weighed, and its yield was calculated (8.39%). LCE was kept under refrigeration until the biological and chemical tests were performed.

### 4.3 UFLC‐QTOF‐MS Analysis

In accordance with the methodology of Lemos et al. [37], the chemical characterization of the LCE was performed by UFLC‐QTOF‐MS using chromatography/MS techniques (*UFLC* Shimadzu Nexera model*, QTOF‐MS* Bruker Compact model electrospray ionization source). The mobile phase used consisted of acidified water, pH = 3, with formic acid (phase A) and methanol (phase B). The injection flow was set to 0.4 mL/min, and the running time to 12 min. The column used was the Kinetex 2.6 µm—C18 ‐ 100A, length 100 mm X 3.0 mm. The chromatographic run began with 40% phase B at 0.01 min, reaching up to 70% phase B at 8.20 min and 95% phase B at 9.70 min, with a subsequent return to 40% phase B at 10.20 min; the run ended in 12 min. The ionization conditions were in positive [M + H]^+^ mode with the following specifications: ion source electrospray voltage of 40 V, a capillary voltage of 4500 V, and a capillary temperature of 220°C. The full scan mass acquisition was performed by scanning from 100 to 1000 *m/z* range.

### 4.4 Animal Procurement

Male BALB/c mice weighing 20–25 g at 30 days of age were obtained from the Federal University of Juiz de Fora Reproduction Biology Center. National Institutes of Health's Ethical Guidelines for the Care and Use of Laboratory Animals were closely followed throughout this study. The Federal University of Juiz de Fora Committee on the Ethics of Animal Experiments approved the study protocol on May 10, 2018 (Protocol Number: 07/2018‐CEUA).

### 4.5 Cytotoxicity Assessment by MTT Assay

The MTT assay was used to evaluate the cytotoxicity of two cell lines: peritoneal macrophages and L929 fibroblasts [38]. Macrophages were obtained from the peritoneal cavity of BALB/c mice, 72 h after intraperitoneal inoculation with 2% thioglycolate (Sigma‐Aldrich Co. Ltd., USA). Macrophages (2x10^5^ cells/well) and murine fibroblasts of the L929 strain (ATCC CCL‐1 NCTC) (RRID: CVCL 0462), obtained commercially from the Rio de Janeiro Cell Bank (5 × 10^3^ cells/well), were seeded into 96‐well microplates and cultured in RPMI medium (Gibco, Thermo Fisher Scientific, USA) for 24 h. Cells were then exposed to LCE at doses varying between 18.75 to 150.00 µg/mL. Vehicle control wells received 0.06% DMSO (Biotec Reagentes Analíticos, Brazil) only. The cells were incubated at 37°C in an environment containing 5% CO_2_ for 48 h. Cells were incubated at 37°C in a 5% CO_2_ atmosphere for 48 h, followed by incubation with MTT solution (5 mg/mL, Sigma‐Aldrich Co. Ltd., USA) for 4 h. Absorbance was measured at 570 nm using a microplate reader (Thermo Scientific Multiskan GO, software 3.2, USA). All experiments were conducted in triplicate.

### 4.6 Evaluation of Anti‐inflammatory Activity

#### 4.6.1 ROS Production in Macrophages Stimulated With LPS and IFN‐γ

Following a 48‐h period, macrophages activated with LPS and IFN‐γ (both from Sigma‐Aldrich Co. Ltd., USA) (2 x 10^5^ cells per well) and treated with LCE at 18.75 ‐ 150 µg/mL had their ROS levels measured. The cells were then labeled with 1 mM of 2′,7′‐Dichlorofluorescin diacetate (H2DCFDA) (Invitrogen, Waltham, MA, USA) [39]. Using a fluorimeter (FLx800) at λex/λem = 485/528 nm, fluorescence (FLx800, BioTek Instruments, Inc., Winooski, VT, USA) values were obtained after 30 min. A positive control group of cells was treated with only LPS and IFN‐γ.

#### 4.6.2 NO Dosage

The Griess method (an indirect NO measurement by nitrite dosage), as outlined by Sun et al. [40], was used to measure NO production. After 48 h of incubation with LCE (18.75–150.00 µg/mL) and stimulation with LPS at 1 µg/mL and IFN‐γ at 1 ng/mL, nitrite levels were assessed in the macrophage culture supernatants. 48 h were spent incubating the cells at 37°C with 5% CO2. Spectrophotometric (Thermo Scientific Multiskan GO, software 3.2, USA) measurements were taken at 540 nm, and the experiment was conducted in triplicate.

#### 4.6.3 Quantification of TNF‐α and IL‐6 Levels

TNF‐α and IL‐6 concentrations were measured using a standard commercially available enzyme‐linked immunosorbent assay (ELISA) kit, following the manufacturer's instructions (R&D Systems, USA). TNF‐α was produced by peritoneal macrophages that were stimulated and treated with LCE at 150.00 µg/mL.

#### 4.6.4 Quantification of TGF‐β Levels

LPS + IFN‐γ (both from Sigma‐Aldrich Co. Ltd., USA) were used to cultivate and excite macrophages that were isolated from the peritoneal cavity of BALB/c mice, in the presence of 150.0 µg/mL of LCE. The supernatants were collected 48 h after stimulation, and TGF‐β concentrations were measured using a commonly used, commercially available enzyme‐linked immunosorbent assay (ELISA) in accordance with the manufacturer's instructions (R&D Systems, USA).

### 4.7 Evaluation of the Accumulation of LBs in Macrophages Stimulated With LPS and IFN‐γ

The accumulation of LBs was assessed in peritoneal macrophages of BALB/c mice in the presence of LCE (150.00 µg/mL) and stimulated with LPS and IFN‐γ (both from Sigma‐Aldrich Co. Ltd., USA). Following this, cells were stained with Nile Red (9‐diethylamino‐5H‐benzo[α]phenoxazine‐ 5‐one), Sigma‐Aldrich Co. Ltd., USA. The protocol followed Basselin et al. [41], with some modifications. Macrophages were cultured for 48 h at 37°C in a 5% CO2 atmosphere after being plated at a density of 2x10^5^ cells per well. The cells were washed with PBS (Gibco, Thermo Fisher Scientific, USA), and subsequently stained with 200 µL of Nile Red (10 µg/mL) in PBS for 20 min at 25°C. Readings were taken using a spectrofluorimeter (FLx800; BioTek Instruments, Inc., Winooski, VT, USA) at 485 and 528 nm for excitation and emission, respectively.

### 4.8 Quantification of PGE2 Levels

Enzyme Immunoassay (EIA) was used to measure the amount of PGE2 produced by peritoneal macrophages activated and treated with LCE at a dosage of 150.00 µg/mL in the supernatant of the cultures, in accordance with the manufacturer's instructions (Cayman Chemical, USA).

### 4.9 Scratch Wound Healing Assay

The healing wound activity of LCE was investigated using a modified method based on Pereira et al. [42]. In this method, 24‐well plates were seeded with 5 × 10^4^ fibroblast cells per well and incubated for 24 h to allow adherence. A linear scratch was made across the cell monolayer using a sterile 200 µL pipette tip, and LCE was applied at concentrations of 18.75 and 37.50 µg/mL, while the control group received fresh DMEM (Gibco, Thermo Fisher Scientific, USA) without treatment. Microscopic images of the scratched areas were taken at 10x magnification using an FLx800 microscope (BioTek Instruments, Inc., Winooski, VT, USA) at 0, 24, and 48 h. The extent of cell migration was determined by measuring the width of the scratch in each image, using Olympus IX51 fluorescence microscopy software for analysis (Olympus Corporation, Tokyo, Japan). The experiment was performed in triplicate, and the migration rate was calculated using the formula:

$$\begin{array}{ccc} \mathrm{Migration}\;\mathrm{rate}\;(\%) & = & \frac{\mathrm{Scratch}\;\mathrm{width}\;(t0)-\mathrm{Scratch}\;\mathrm{width}\;(\mathrm{tf})}{\mathrm{Scratch}\;\mathrm{width}\;(t0)}\\ & & \times 100 \end{array}$$

The initial scratch width (t0) corresponds to measurements taken at 0 h, and the final scratch width (tf) corresponds to measurements taken at 24 or 48 h.

### 4.10 Statistical Analysis

Using the program GraphPrism 5.0, statistical analysis was carried out using the analysis of variance test and then Dunnett's Multiple Comparison Test. The Student's t‐test was used in a few instances. *p* < 0.05 values were regarded as significant. The findings were presented as the average ± standard deviation.

## Author Contributions

The study design, study mentorship, and manuscript critical editing were supplied by Rodrigo L. Fabri and Elaine S. Coimbra The study design was influenced by Matheus T. Branca, Natasha S. Mayrink, Lívia R. Gamarano, P.L.P., João Pedro R. C. Bastos, Ana Julia de Almeida, Matheus T. Branca, Natasha S. Mayrink, Ari S. de O. Lemos, and Matheus T. Branca conducted experiments and collected and examined the data. Each author authorized the completed version of the manuscript after contributing in part to its writing and editing.

## Conflicts of Interest

The authors declare no conflicts of interest.

## Funding

Grants were used to fund this effort from Conselho Nacional de Desenvolvimento Científico e Tecnológico (CNPq, Brazil—grant number: 408700/2021‐1) and Fundação de Amparo à Pesquisa do Estado de Minas Gerais (grant numbers: APQ‐01357‐21, APQ‐01646‐23, and RED‐00198‐23). A.S.O.L. was a grant recipient of Coordenação de Aperfeiçoamento de Pessoal de Nível Superior (CAPES, Brazil). L.M.C., E.S.C., and R.L.F. are grant recipients of CNPq.

## Data Availability

The publication contains all the data from this work. The plant species' use license is registered with SISGEN/Brazil.—A18AB08.
